# Greater mortality variability in the United States in comparison with peer countries

**DOI:** 10.4054/demres.2020.42.36

**Published:** 2020-06-09

**Authors:** Richard G. Rogers, Robert A. Hummer, Justin M. Vinneau, Elizabeth M. Lawrence

**Affiliations:** 1 Department of Sociology and Population Program, Institute of Behavioral Science (IBS), University of Colorado Boulder, USA.; 2 Department of Sociology and Carolina Population Center, University of North Carolina at Chapel Hill, USA.; 3 Department of Sociology and Population Program, Institute of Behavioral Science (IBS), University of Colorado Boulder, USA.; 4 Department of Sociology, University of Nevada, Las Vegas, USA.

## Abstract

**BACKGROUND:**

Over the past several decades, US mortality declines have lagged behind other high-income countries. However, scant attention has been devoted to how US mortality variability compares with other countries.

**OBJECTIVE:**

We examine trends in mortality and mortality variability in the US and 16 peer countries from 1980 through 2016.

**METHODS:**

We employ the Human Mortality Database and demographic techniques – with a focus on patterns in the interquartile (IQR), interdecile (IDR), and intercentile (ICR) ranges of survivorship – to better understand US mortality and mortality variability trends in comparative perspective.

**RESULTS:**

Compared to other high-income countries, the US: (1) mortality ranking has slipped for nearly all age groups; (2) is losing its old age mortality advantage; (3) has seen growth in relative age-specific mortality gaps from infancy through midlife; and (4) exhibits greater concentrations of deaths from infancy through adulthood, resulting in much greater mortality variability.

**CONCLUSIONS:**

We contribute to calls for renewed attention to the relatively low and lagging US life expectancy. The ICR draws particular attention to the comparatively high US early and midlife mortality.

**CONTRIBUTION:**

We find comparatively high variability in US mortality. Further reductions in early and midlife mortality could diminish variability, reduce years of potential life lost, and increase life expectancy. Consistent with previous research, we encourage policymakers to focus on reducing the unacceptably high early and midlife mortality in the US. And we urge researchers to more frequently monitor and track mortality variation in conjunction with mortality rates and life expectancy estimates.

## 1. Introduction

Compared to other high-income countries, US age-specific mortality rates are higher and life expectancy is lower (Avendano and Kawachi 2014; Ho and Hendi 2018; Khan et al. 2018; National Research Council and Institute of Medicine [NRC/IoM] 2013). Unfortunately, US life expectancy at birth was the same in 2018 as in 2010; it declined from 2014 through 2017 (Kochanek et al. 2019) and increased just 0.1 years between 2017 and 2018 (Xu et al. 2020). To better understand how and why the United States lags behind other high-income countries and to highlight new features of the US mortality disadvantage, we compare trends in age-specific mortality and mortality variability from 1980 through 2016 in the United States to those in 16 peer countries.

## 2. Background

### 2.1 Motivations for studying mortality variability

International comparisons of mortality variability, both at one point in time and over time, can inform when and how the US mortality disadvantage emerged and how it persists, and can complement such key demographic measures as life expectancy and age-specific mortality rates (Acciai and Firebaugh 2019; Shkolnikov et al. 2011; Vaupel, Zhang, and van Raalte 2011; Wilmoth and Horiuchi 1999). Mortality variability is particularly important given recent research highlighting the contribution of deaths at younger ages to the US mortality disadvantage (Ho 2013; Ho and Hendi 2018; Vaupel, Zhang, and van Raalte 2011). More person-years are affected and the years of potential life lost are far greater when people die in early rather than in later life. Among high-income peer countries, the United States has relatively low mortality at older ages (e.g., ages 80 and above), but higher mortality from birth to age 75 (Ho and Preston 2010; Manton and Vaupel 1995; NRC/IoM 2013). Furthermore, recent studies have demonstrated that the United States has especially high mortality at ages below 50 (Ho 2013; Ho and Hendi 2018). Thakrar and colleagues (2018) find that compared to children ages 0–19 in 19 Organization for Economic Cooperation and Development (OECD) countries, US children have experienced higher mortality since the 1980s, with widening disparities over time. Indeed, for the 2001–2010 decade and compared to 19 other OECD countries, the mortality rate for US infants was 76% higher, and the mortality rate for US children aged 1–19 was 55% higher (Thakrar et al. 2018).

Because changes in mortality variability for the United States relative to other high-income countries over time may also inform the current US mortality disadvantage, we examine the interquartile (IQR), interdecile (IDR), and intercentile (ICR) ranges of survivorship, which are valuable measures of variation (van Raalte, Sasson, and Martikainen 2018; Tuljapurkar and Edwards 2011; Wilmoth and Horiuchi 1999). The IQR is preferred over alternative measures of mortality variability such as the standard deviation or the Gini coefficient because it is easy to calculate, understand, and interpret (Wilmoth and Horiuchi 1999). The IQR indicates the country-specific difference between the 25^th^ and 75^th^ percentiles in survivorship, the IDR provides the difference between the 10^th^ and 90^th^ percentiles, and the ICR reports the difference between the 1^st^ and 99^th^ percentiles. Larger ranges in these three measures indicate more variability and uncertainty, whereas smaller ranges signal greater regularity in lifespans (Vaupel, Zhang, and van Raalte 2011). Larger ranges in the measures are often but not necessarily the result of greater concentrations of deaths at younger ages (van Raalte, Sasson, and Martikainen 2018).

The lower bounds of the IQR, IDR, and ICR indicate how well countries prevent mortality early in life. Many early life deaths are due to external causes and therefore could be averted. Thus, with fewer deaths at younger ages, countries generally exhibit more mortality compression (i.e., less variability). The upper bounds indicate how well countries address senescent mortality. Many of these deaths are due to chronic and degenerative diseases and are generally more difficult to reduce than deaths at younger ages. Thus, the ranges could also expand if countries experience mortality improvement in older ages.

The IQR, IDR, and ICR offer unique, complementary information. The IQR has the narrowest range and, given the typical left-skewed age distribution of deaths in high-income countries (Rogers et al. 2019), provides insight on mortality variability in older adulthood. The IDR has a wider range and thus insight into the distribution of deaths ranging from middle to older adulthood. The ICR has the largest range and therefore valuable insight regarding mortality in early life and very old ages. Differences and similarities in the patterns and trends of these three measures may shed light on the processes and age groups contributing most to the US mortality disadvantage.

### 2.2 Research aims

We build on previous studies to document and compare mortality and mortality variability trends for the United States and 16 peer countries. We first present age-specific mortality rates and the distribution of deaths and then turn to measures of mortality variability (IQR, IDR, and ICR).

## 3. Methods

### 3.1 Comparison countries

We compare the United States to 16 other high-income countries: Australia, Austria, Canada, Denmark, Finland, France, Germany, Italy, Japan, Netherlands, Norway, Portugal, Spain, Sweden, Switzerland, and the United Kingdom (for similar comparisons, see Ho [2013], Ho and Preston [2010], and Thakrar et al. [2018]). OECD has identified them as the most comparable to the United States based on similar levels of wealth and economic development (Ho 2013).

### 3.2 Data and methods

We examine life table values between 1980 and 2016^5^ from the public online Human Mortality Database (HMD 2020). We analyze trends in age-specific mortality for each of the 17 countries in five-year periods. We also report the percentage differences in age-specific mortality probabilities (q_x_) between the United States and the average of the comparison countries within each year. Positive percentages indicate a higher US mortality probability; negative percentages indicate lower US mortality probabilities. We then highlight the distributions of death (d_x_) across age in the most recent year of available data, 2016. Finally, we examine single-year periods and age groups to calculate the IQR, IDR, and the ICR of survivorship (l_x_). Based on a radix of 100,000, the IQR measures the range between the age of death for the 1^st^ and 3^rd^ quartiles (l_x_ values of 25,000 and 75,000, respectively) and represents the range where the middle 50% of deaths fall relative to age (Siegel 2012; Wilmoth and Horiuchi 1999). The IDR determines the ages at which l_x_ values reach 10,000 and 90,000, and the ICR determines the ages at which l_x_ values reach 1,000 and 99,000.

## 4. Results

Between 1980 and 2016, US age-specific mortality rankings worsened (Table 1). For example, for the 5–9 age group, the United States ranked 10^th^ (out of 16 countries) in 1980, 14^th^ (out of a total of 17 countries) in 1990, and last in 2014 and 2016. Across time, more US age groups have moved to last place (shaded blue). The number of US age groups in the worst ranking was just 1 in 1985 and in 1990, 3 in 2000, 13 in 2010, and 17 in 2016. In 2016 the United States ranked last for all age groups under 80. Conversely, the United States ranks favorably at advanced ages. For example, in 2016, the United States had the lowest or second lowest mortality probability at ages 90 and above. However, US rankings at the older ages have also slipped.

**Figure 1:**
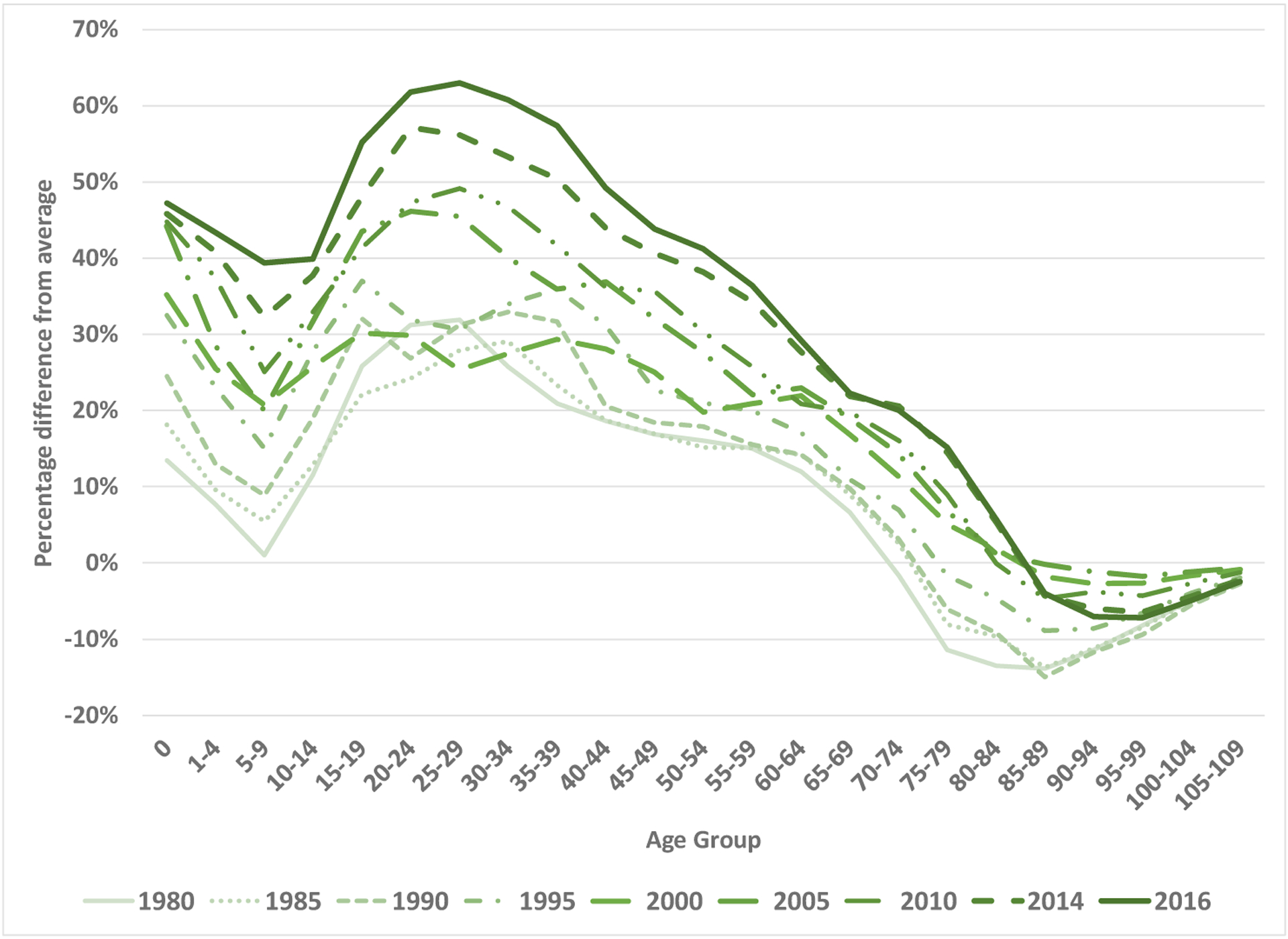
Age-specific percentage differences in mortality probabilities between the United States and the average of 16 peer countries, 1980–2016 *Note*: The average for peer countries excludes Germany in 1980, and excludes Italy and Portugal in 2016. *Source*: Derived from the Human Mortality Database (2020).

The largest percentage differences in mortality probabilities between the United States and its peers between years 1980 and 2016 are in recent periods and at younger ages (see Figure 1). For example, compared to the average mortality probabilities in peer countries, the US mortality probability for ages 25–29 was 28% higher in 1985, but 63% higher in 2016. In 2016 the percentage gap in age-specific mortality probabilities between the United States and the average of its peers was 47% for ages 0–1, 39% for ages 5–9, 55% for ages 15–19, and 62% for ages 20–24. By contrast, the mortality probabilities at ages 85 and above continue to favor the United States in 2016 relative to the average of its peers by 2% to 8%, depending on the five-year age group.

**Figure 2:**
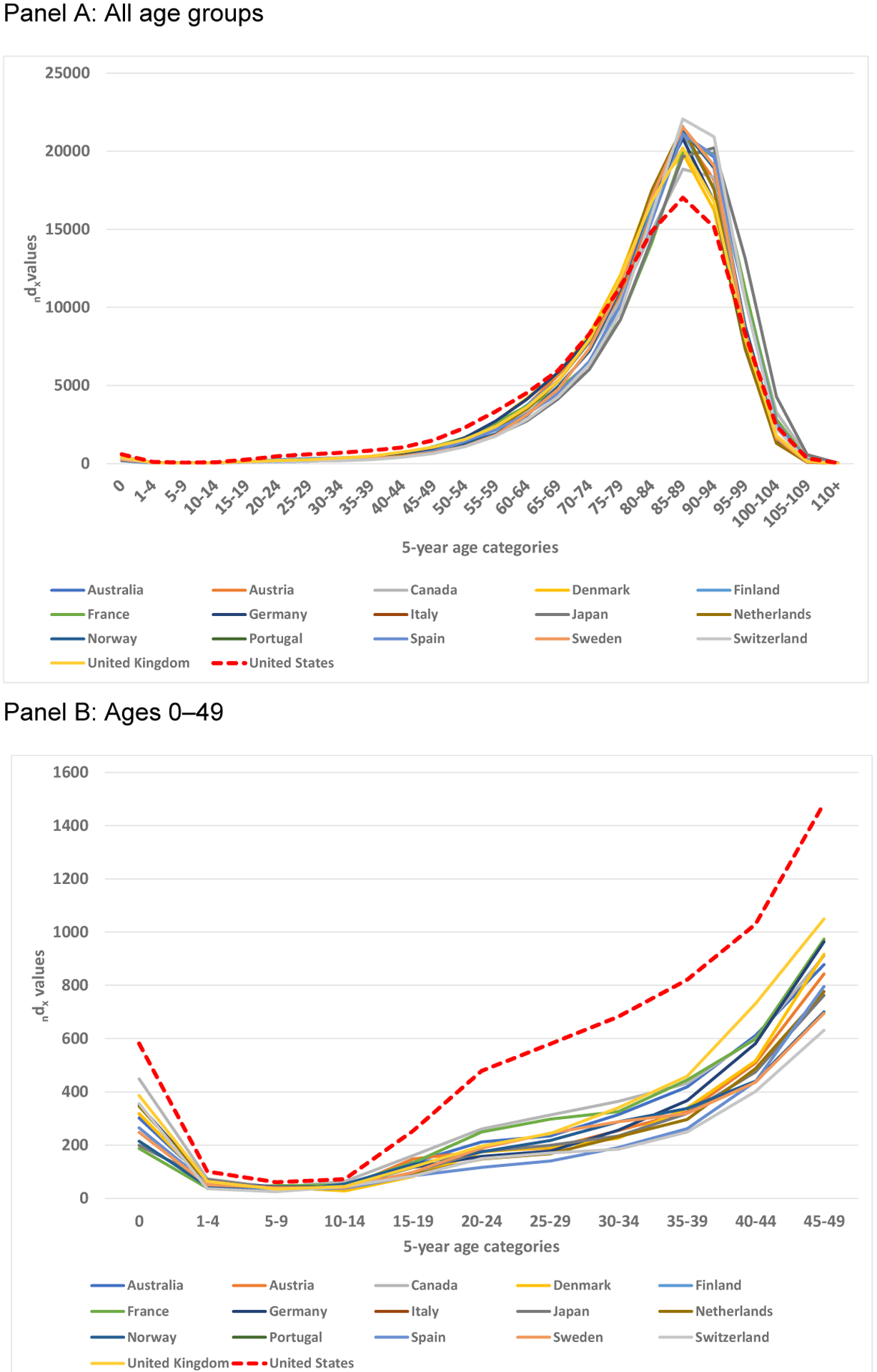
Distributions of deaths (_n_d_x_) by country, 2016 *Source*: Derived from the Human Mortality Database (2020).

Figure 2, Panel A shows that the US distribution of deaths (d_x_) in 2016 peaks in the same age group (85–89) as most other countries but has a shorter height and wider spread. Whereas most other countries peak close to and often above 20,000 deaths (with a radix of 100,000) for the modal five-year age group, the United States peaks around 17,000 deaths. This comparison, along with the heightened left tail of the distribution, shows that the United States experiences comparatively more deaths earlier in life. The scale obscures the larger numbers of deaths at younger ages. Panel B underscores the large disparity between the United States and other countries in deaths among individuals younger than 50 years of age, especially during infancy and in the age range of 15–49.

**Figure 3:**
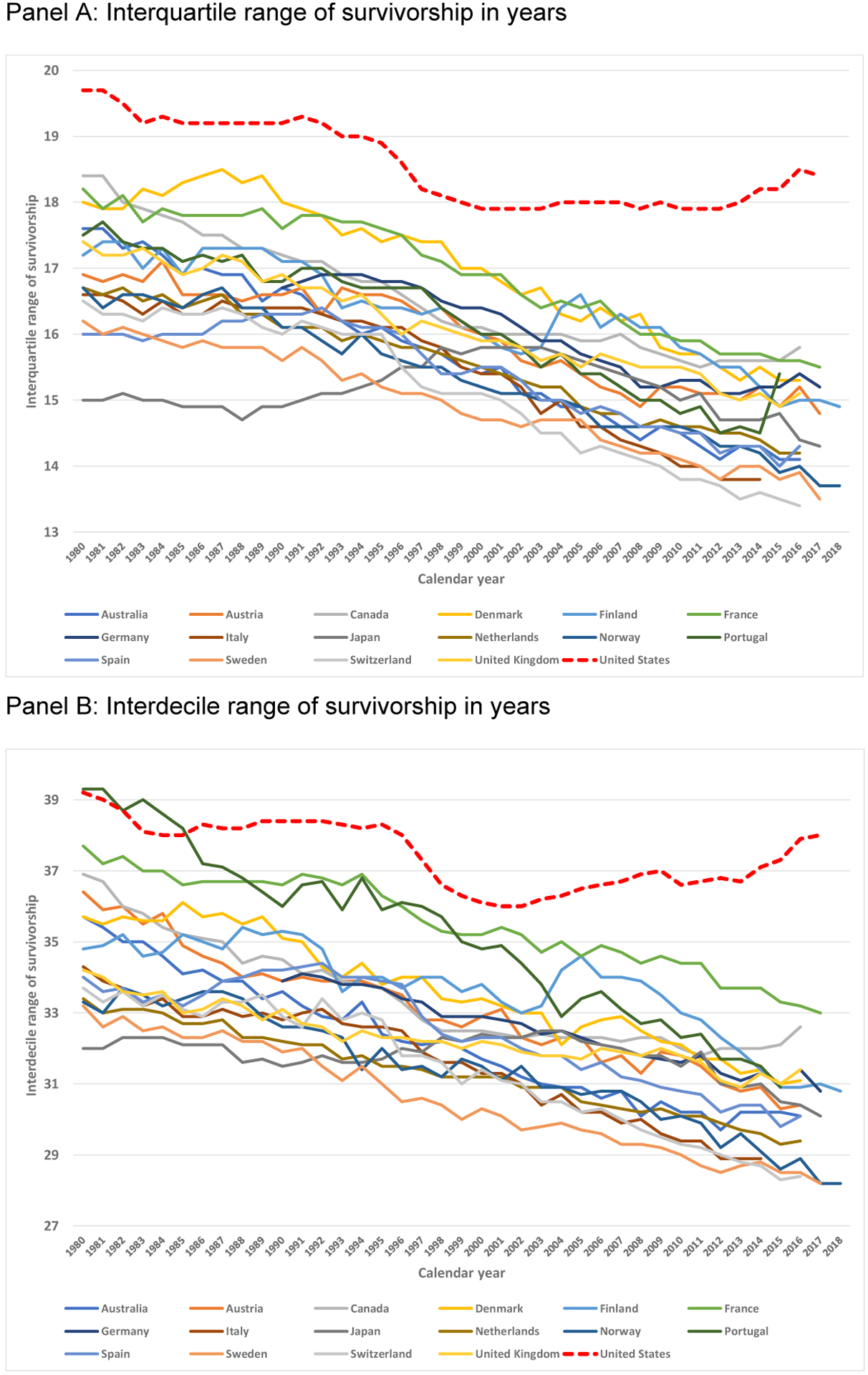

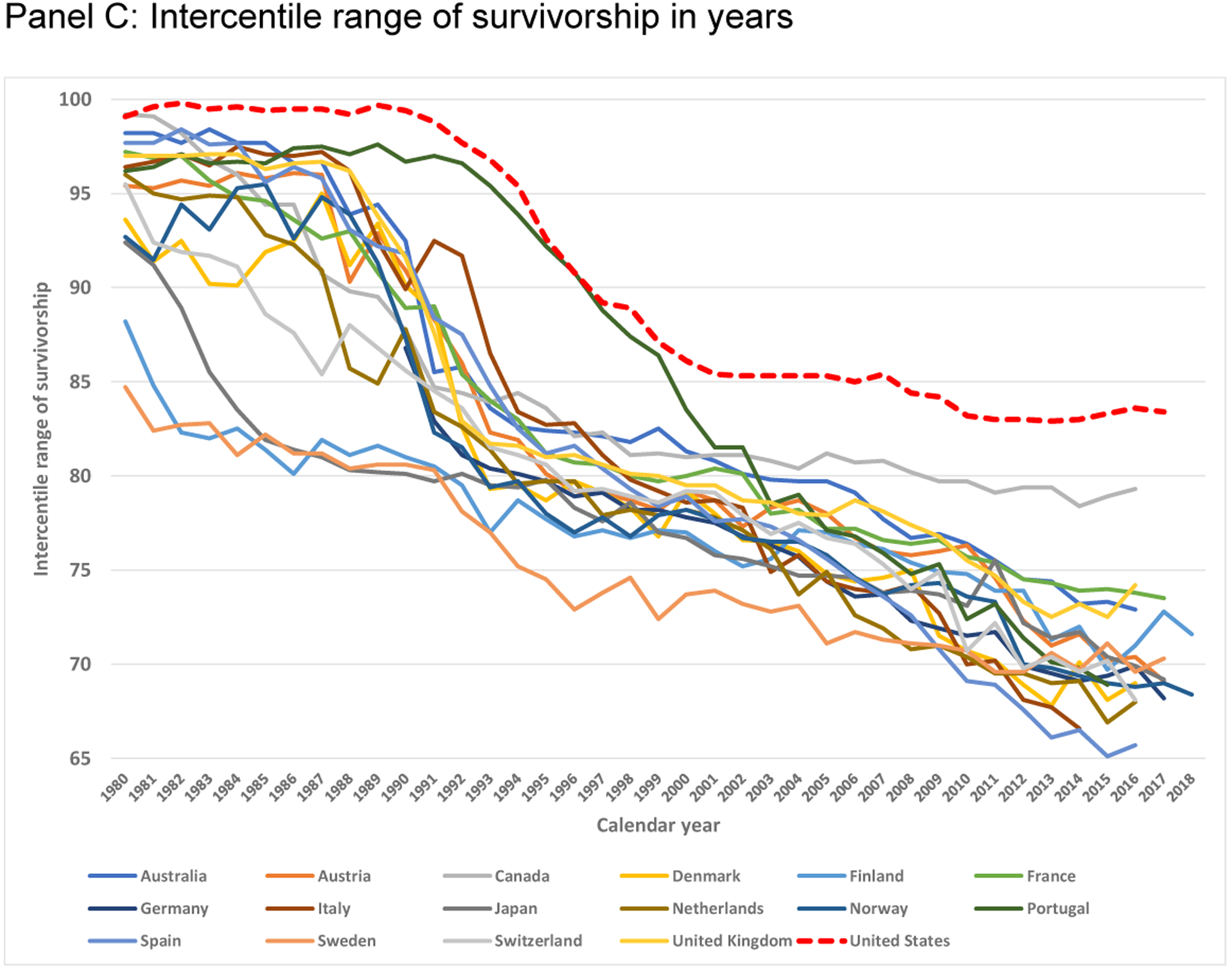
High US interquartile, interdecile, and intercentile ranges of survivorship relative to peer countries, 1980–2018 *Notes*: Total of countries included in 1980 is 16, with Germany excluded. Total of countries included in 2016 is 15, with Italy and Portugal excluded. The figure includes trends through 2018 for countries with available data (Finland and Norway). *Source*: Derived from the Human Mortality Database (2020).

The IQRs display relatively high US mortality variability in adulthood. Compared to the 16 peer countries, US IQRs are substantially higher for every year since 1980, with greater concentrations of deaths at younger ages (see Table 2, Panel A, and Figure 3, Panel A). For example, in 2016 the US lower bound was 71.9 years and the upper bound was 90.3 years, producing an IQR of 18.4, a value 2.6 years (16%) higher than that of the next country, Canada, and 5.0 years (37%) higher than the country with the smallest IQR, Switzerland. While other countries reduced their IQRs over time, the US IQR remained relatively stagnant in the 2000s, and has increased since 2012. This flattening and recent increase is a result of the United States continuing to experience mortality reductions at older ages but mortality stagnation in early and midlife, thus increasing its IQR range while most IQRs for other countries are contracting due to mortality improvements at both younger and older ages. The year 2016 marks the greatest gap since 1980 between the IQR of the United States and that of the next closest country. The lower quartile appears to drive this gap, distinguishing the United States from peer countries. In 2016 the upper quartile bound lies between 90.0 and 93.0 for nearly every country, including the United States; however, the lower quartile bound is 71.9 years for the United States but between 74.7 and 78.7 for other countries.

The findings for the IDR and ICR are consistent with those of the IQR, but impart additional detail. The upper decile and centile bounds for the United States compare favorably to those of the 16 comparison countries, but the United States has experienced stagnation in the lower decile and centile bounds. By 2016 the United States was the only country with a lower decile bound below age 60, lagging 5.7 years behind the closest nations of France and Germany (Table 2, Panel B).

In 1980 few countries exhibited ICR values of less than 90 and most had lower centile bounds below the age of 1. By 1990 nearly every country exhibited a lower bound above 7 years old. The United States was a clear exception, with a lower centile bound of 1.5. Despite this great lag, the United States was not alone: Portugal’s lower bound was 0.9 years. But by 2000 Portugal had surpassed the United States in its lower centile bound and by 2014 exhibited a lower centile bound of 31.3 years. In 2016 the United States exhibited a lower centile bound of 19.3, a difference of 4.8 years from the next lower value of 24.1 years for Canada, and 17.4 years for the country with the highest value of 36.7, Spain.

Sex-specific results across nations reveal similar patterns. The United States is characterized by higher IQR, IDR, and ICR ranges for both males and females (see Appendix Figure A), and the disparity between the United States and the other countries has been increasing since 1980 for all three measures of variability. The reason for the greater and increasing sex-specific dispersions in the United States is the much lower IQR, IDR, and ICR lower bounds for both US women and men in comparison to peer countries.

## 5. Discussion

Compared to other high-income countries, the United States has higher mortality and lower life expectancy, with a ranking that has slipped over the past several decades (see Avendano and Kawachi 2014; Ho and Hendi 2018; Ho and Preston 2010; NRC/IoM 2013). In 2016, among the high-income peer countries we examined, US age-specific mortality probabilities were highest for each five-year age group below age 80; moreover, rankings for ages 80–89 have slid over time. For instance, the age group 80–84 dropped from 2^nd^ to 10^th^ place between 1980 and 2016. Furthermore, between 2010 and 2016, age-specific mortality percentage differences worsened between the United States and other high-income countries among all ages under 85.

Most centrally, our results demonstrate the importance of documenting patterns and trends in variability in age at death across countries. Some of the most striking differences between the United States and peer countries are in infant, late adolescent, and young adult mortality. Indeed, compared to peer countries in 2016, the United States experiences age-specific mortality probabilities that are at least 60% higher for ages 20–24 and 25–29. The United States is the only country we examined where 1% of the deaths occur among individuals younger than 20 years old (based on the ICR), where 10% of deaths still occur before age 60 (based on the IDR), and where 25% of deaths continue to occur by age 72 (based on the IQR). All three of these measures illustrate the need for the United States to focus efforts on reducing its mortality rates up to age 72, with perhaps the greatest attention on the youngest ages. By contrast, regarding the upper bounds of the IQR, IDR, and ICR the United States exhibits few differences in comparison to its peer countries.

Greater mortality variation within a country could create social, behavioral, and psychological uncertainties for individuals. Future research could examine whether Americans express greater uncertainty in planning for education, work, marriage, childbearing, and retirement. Further, greater mortality variation at the national level challenges government planning for old age programs such as Social Security and Medicare. Mortality variation is an important demographic tool, especially when paired with other measures. Thus, we encourage countries to monitor mortality variation and upper and lower bounds in addition to mortality rates and life expectancy (van Raalte, Sasson, and Martikainen 2018; Tuljapurkar and Edwards 2011; Wilmoth and Horiuchi 1999).

Continued monitoring will be especially vital amid the spread of the new coronavirus, COVID-19. The pandemic will undoubtedly increase mortality and reduce life expectancy throughout the world (see Olshansky et al. 1997). As of the beginning of June 2020, compared to peer countries the United States has had a slower and less effective response; less federal leadership, coordination, and oversight; and less public support for and compliance with community public health efforts to test and treat COVID-19 cases (Edwards 2020). Thus, the United States is likely to see a more pronounced spike in COVID-19 cases and greater strains on the health infrastructure, leading to a declining upper bound and higher mortality among the elderly. The reduction in the upper bound may, then, reduce variability in US mortality, while also reducing life expectancy. Thus, tracking both US life expectancy and mortality variability will be important for a continued comprehensive understanding of mortality change.

## Supplementary Material

DR_Supplementary_Material

## Figures and Tables

**Table 1:** Age-specific mortality (_n_q_x_) ranking of the United States compared to 15 or 16 peer countries, selected years, 1980–2016

Age	1980*	1985*	1990	1995	2000	2005	2010	2014	2016^†^
0	13	14	16	17	17	17	17	17	15
1–4	12	14	16	15	16	17	17	17	15
5–9	10	12	14	16	16	15	16	17	15
10–14	13	14	16	16	16	17	17	17	15
15–19	14	15	16	17	17	17	17	17	15
20–24	16	15	16	16	16	17	17	17	15
25–29	16	15	16	16	16	17	17	17	15
30–34	15	16	17	15	16	16	17	17	15
35–39	15	15	17	17	16	16	17	17	15
40–44	14	13	16	17	16	17	17	17	15
45–49	14	13	15	17	17	17	17	17	15
50–54	16	15	15	17	16	17	17	17	15
55–59	15	15	16	16	16	17	17	17	15
60–64	13	14	15	16	16	17	17	17	15
65–69	11	13	14	15	16	16	16	17	15
70–74	9	10	10	14	15	16	16	17	15
75–79	2	5	6	9	12	13	15	16	15
80–84	2	3	3	8	9	11	8	12	10
85–89	2	2	1	2	8	7	6	6	5
90–94	2	1	2	2	5	7	4	4	2
95–99	1	1	1	2	3	5	3	2	2
100–104	1	1	1	1	3	4	2	2	1
105–109	1	1	1	1	3	3	2	1	1

* Total of countries included is 16; Germany is missing.

† Total of countries included is 15; Italy and Portugal are missing.

Note: Blue solid shaded areas indicate where the United States ranks last; light green shaded areas indicate where the United States ranks first.

Source: Derived from the Human Mortality Database (2020).

**Table 2:** United States has wider interquartile, interdecile, and intercentile ranges for survivorship, with younger lower bounds

	1980*		1990		2000		2010		2014		2016†	
A. Interquartile Range for Survivorship												
	[Q3; Q11	IQR	[Q3; Q1]	IQR	[Q3; Q1]	IQR	[Q3; Q1]	IQR	[Q3; Q1]	IQR	[Q3; Q1]	IQR
Australia	[85.5; 67.9]	17.6	[87.4; 70.7]	16.7	[89.4; 74.0]	15.4	[91.4; 76.9]	14.5	[91.7; 77.4]	14.3	[91.9; 77.8]	14.1
Austria	[83.7; 66.8]	16.9	[86.1; 69.5]	16.6	[88.2; 72.2]	16.0	[89.9; 74.7]	15.2	[90.7; 75.7]	15.2	[90.8; 75.6]	15.2
Canada	[86.5; 68.1]	18.4	[88.0; 70.8]	17.2	[89.1; 73.0]	16.0	[91.1; 75.7]	15.6	[91.6; 76.0]	15.6	[92.0; 76.2]	15.8
Denmark	[85.1; 67.1]	18.0	[85.7; 67.7]	18.0	[87.0; 70.0]	17.0	[88.7; 73.0]	15.7	[90.0; 74.5]	15.5	[90.1; 74.8]	15.3
Finland	[84.2; 67.0]	17.2	[85.6; 68.5]	17.1	[87.7; 71.7]	16.0	[89.8; 74.0]	15.8	[90.4; 75.2]	15.2	[90.5; 75.5]	15.0
France	[85.7; 67.5]	18.2	[87.9; 70.3]	17.6	[89.6; 72.7]	16.9	[91.4; 75.7]	15.9	[92.2; 76.5]	15.7	[92.1; 76.5]	15.6
Germany	-	-	[85.7; 69.0]	16.7	[88.0; 71.6]	16.4	[89.5; 74.2]	15.3	[90.2; 75.0]	15.2	[90.1; 74.7]	15.4
Italy	[84.7; 68.1]	16.6	[87.2; 70.8]	16.4	[89.2; 73.8]	15.4	[90.8; 76.8]	14.0	[91.5; 77.7]	13.8	-	-
Japan	[85.8; 70.8]	15.0	[88.4; 73.5]	14.9	[90.9; 75.1]	15.8	[92.4; 77.4]	15.0	[92.9; 78.2]	14.7	[93.2; 78.7]	14.4
Netherlands	[86.1; 69.4]	16.7	[86.9; 70.8]	16.1	[87.6; 72.1]	15.5	[89.8; 75.2]	14.6	[90.5; 76.1]	14.4	[90.3; 76.1]	14.2
Norway	[86.0; 69.3]	16.7	[86.6; 70.5]	16.1	[88.2; 73.0]	15.2	[90.1; 75.5]	14.6	[90.9; 76.7]	14.2	[91.1; 77.1]	14.0
Portugal	[83.7; 66.2]	17.5	[85.3; 68.5]	16.8	[87.2; 71.2]	16.0	[89.3; 74.5]	14.8	[90.2; 75.7]	14.5	-	-
Spain	[85.9; 69.9]	16.0	[87.5; 712]	16.3	[89.2; 73.7]	15.5	[91.1; 76.6]	14.5	[91.8; 77.5]	14.3	[91.9; 77.6]	14.3
Sweden	[85.8; 69.6]	16.2	[87.3; 71.7]	15.6	[88.8; 74.1]	14.7	[90.2; 76.1]	14.1	[90.9; 76.9]	14.0	[91.0; 77.1]	13.9
Switzerland	[86.1; 69.6]	16.5	[87.6; 71.6	16.0	[89.4; 74.3]	15.1	[91.0; 77.2]	13.8	[91.7; 78.1]	13.6	[91.9; 78.5]	13.4
United Kingdom	[84.2; 66.8]	17.4	[85.9; 69.0]	16.9	[87.6; 71.7]	15.9	[90.0; 74.5]	15.5	[90.5; 75.4]	15.1	[90.4; 75.3]	15.1
**United States**	**[85.9; 66.2]**	**19.7**	**[87.2; 68.0]**	**19.2**	**[87.9; 70.0]**	**17.9**	**[89.9; 72.0]**	**17.9**	**[90.3; 72.1]**	**18.2**	**[90.3; 71.9]**	**18.4**
B. Interdecile Range for Survivorship												
	[D9;D1]	IDR	[D9;D1]	IDR	[D9;D1]	IDR	[D9;D1]	IDR	[D9;D1]	IDR	[D9;D1]	IDR
Australia	[91.2; 55.5]	35.7	[92.6; 59.0]	33.6	[94.1; 62.4]	31.7	[95.6; 65.4]	30.2	[96.0; 65.8]	30.2	[96.2; 66.1]	30.1
Austria	[88.9; 52.5]	36.4	[90.9; 57.0]	33.9	[92.7; 59.8]	32.9	[94.4; 62.6]	31.8	[94.9; 64.0]	30.9	[95.0; 64.6]	30.4
Canada	[92.2; 55.3]	36.9	[93.4; 58.9]	34.5	[94.1; 61.6]	32.5	[95.7; 63.7]	32.0	[96.2; 64.2]	32.0	[96.7; 64.1]	32.6
Denmark	[90.6; 54.9]	35.7	[91.1; 56.0]	35.1	[92.3; 58.9]	33.4	[93.6:61.5]	32.1	[94.6; 63.2]	31.4	[94.7; 63.6]	31.1
Finland	[89.5; 54.7]	34.8	[90.7; 55.4]	35.3	[92.4; 58.6]	33.8	[94.3; 61.3]	33.0	[94.7; 63.3]	31.4	[94.8; 63.9]	30.9
France	[90.9; 53.2]	37.7	[92.8; 56.2)	36.6	[94.2; 59.0]	35.4	[96.0:61.6]	34.4	[96.7; 63.0]	33.7	[96.4; 63.2]	33.2
Germany	-	-	[90.6; 56.7]	33.9	[92.8; 59.9]	32.9	[93.9; 62.3]	31.6	[94.6; 63.3]	31.3	[94.6; 63.2]	31.4
Italy	[89.9; 55.6]	34.3	[92.1; 59.3]	32.8	[93.8; 62.5]	31.3	[95.3:65.9]	29.4	[95.8; 66.9]	28.9	-	-
Japan	[90.6; 58.6]	32.0	[93.0; 61.5]	31.5	[95.7; 63.3]	32.4	[96.9; 65.4]	31.5	[97.5; 66.5]	31.0	[97.6; 67.2]	30.4
Netherlands	[91.4; 58.0]	33.4	[91.9; 59.7]	32.2	[92.4; 61.2]	31.2	[94.3; 64.2]	30.1	[94.8; 65.2]	29.6	[94.6; 65.2]	29.4
Norway	[91.1; 57.8]	33.3	[91.7; 59.1]	32.6	[92.9; 61.4]	31.5	[94.2:64.1]	30.1	[95.1; 66.0]	29.1	[95.3; 66.4]	28.9
Portugal	[89.1; 49.8]	39.3	[90.3; 54.3]	36.0	[92.1; 57.3]	34.8	[93.9:61.6]	32.3	[94.6; 63.1]	31.5		
Spain	[91.0; 57.0]	34.0	[92.3; 58.1]	34.2	[93.8; 61.5]	32.3	[95.5; 64.7]	30.8	[96.2; 65.8]	30.4	[96.2; 66.1]	30.1
Sweden	[90.9; 57.7]	33.2	[92.1; 60.2]	31.9	[93.4; 63.1]	30.3	[94.5; 65.5]	29.0	[95.1:66.3]	28.8	[95.1; 66.6]	28.5
Switzerland	[91.0; 57.3]	33.7	[92.3; 59.4]	32.9	[93.8; 62.4]	31.4	[95.2; 65.9]	29.3	[95.7; 67.0]	28.7	[95.9; 67.5]	28.4
United Kingdom	[89.8; 55.6]	34.2	[91.5; 58.4]	33.1	[92.8; 60.6]	32.2	[94.7; 62.9]	31.8	[95.1; 63.8]	31.3	[95.0; 63.6]	31.4
**United States**	**[91.8; 52.6]**	**39.2**	**[93.0; 54.6]**	**38.4**	**[93.1; 57.0]**	**36.1**	**[94.3; 57.7]**	**36.6**	**[95.4; 58.3]**	**37.1**	**[95.5; 57.5]**	**38.0**
C. Intercentile Range for Survivorship												
	[C99;C1]	ICR	[C99;C1]	ICR	[C99;C1]	ICR	[C99;C1]	ICR	[C99;C1]	ICR	[C99;C1]	ICR
Australia	[99.2; 1.0]	98.2	[100.1; 7.6]	92.5	[100.8; 19.5]	81.3	[101.8; 25.4]	76.4	[102.2; 29.0]	73.2	[102.4; 29.5]	72.9
Austria	[96.1; 0.7]	95.4	[98.0; 7.1]	90.9	[99.3; 20.1]	79.2	[100.6; 24.3]	76.3	[101.1; 29.5]	71.6	[101.2; 30.8]	70.4
Canada	[100.2; 1.0]	99.2	[101.1; 13.4]	87.7	[101.2; 20.2]	81.0	[102.4; 22.7]	79.7	[102.9; 24.5]	78.4	[103.4; 24.1]	79.3
Denmark	[97.9; 4.3]	93.6	[98.6; 8.5]	90.1	[99.6; 20.4]	79.2	[100.7; 30.0]	70.7	[101.5; 31.4]	70.1	[101.3; 32.3]	69.0
Finland	[97.1; 8.9]	88.2	[98.0; 17.0]	81.0	[99.1; 22.1]	77.0	[100.7; 25.9]	74.8	[100.8; 28.8]	72.0	[100.9; 29.9]	71.0
France	[98.2; 1.0]	97.2	[99.7; 10.8]	88.9	[100.9; 20.9]	80.0	[102.4; 26.7]	75.7	[103.0; 29.1]	73.9	[102.6; 28.8]	73.8
Germany	-	-	[97.7; 10.9]	86.8	[99.6; 21.8]	77.8	[100.4; 28.9]	71.5	[100.9; 31.8]	69.1	[100.6; 31]	69.9
Italy	[97.1; 0.7]	96.4	[99.1; 9.2]	89.9	[100.6; 22.0]	78.6	[101.7; 31.7]	70.0	[102.1; 35.5]	66.6	-	-
Japan	[97.6; 5.2]	92.4	[99.8; 19.7]	80.1	[102.5; 25.8]	76.7	[103.5; 30.4]	73.1	[104.0; 32.3]	71.7	[103.9; 34]	69.9
Netherlands	[98.9; 2.9]	96.0	[99.1; 11.3]	87.8	[99.2; 21.0]	78.2	[100.8; 30.4]	70.4	[101.1; 32.0]	69.1	[100.7; 32.7]	68.0
Norway	[98.7; 6.0]	92.7	[98.8; 11.5]	87.3	[99.6; 21.4]	78.2	[100.8; 27.2]	73.6	[101.4; 32.0]	69.4	[101.4; 32.6]	68.8
Portugal	[96.6; 0.4]	96.2	[97.6; 0.9]	96.7	[99.3; 15.8]	83.5	[100.6; 28.21	72.4	[100.9; 31.3]	69.8	-	-
Spain	[98.6; 0.9]	97.7	[99.2; 7.4]	91.8	[100.6; 21.7]	78.9	[101.9; 32.8]	69.1	[102.5; 36.0]	66.5	[102.4; 36.7]	65.7
Sweden	[98.2; 13.5]	84.7	[99.1; 18.5]	80.6	[99.9; 26.2]	73.7	[100.7; 30.0]	70.7	[101.2; 31.5]	69.7	[101.2; 31.6]	69.6
Switzerland	[98.0; 2.5]	95.5	[99.0; 13.4]	85.6	[100.3; 21.1]	79.2	[101.2; 30.5]	70.7	[101.7; 32.1]	69.6	[101.9; 33.8]	68.1
United Kingdom	[97.8; 0.8]	97.0	[99.3; 7.7]	91.6	[100.0; 20.5]	79.5	[101.4; 25.9]	75.7	[101.8; 28.6]	73.2	[101.6; 27.4]	74.2
**United States**	**[99.9; .8]**	**99.1**	**[100.9; 1.5]**	**99.4**	**[100.4; 14.3]**	**86.1**	**[101.9; 18.7]**	**83.2**	**[102.6; 19.6]**	**83.0**	**[102.7; 19.3]**	**83.4**

* Total countries included = 16 - Germany Missing;

† Total countries included = 15 - Italy and Portugal Missing

Source: Derived from the Human Mortality Database (2019). 100.9; 1.5]

## Footnotes

5 Data for Germany became available in 1990. Data for 2016 are available for all countries except Italy and Portugal. Figure 3 presents mortality variability through 2018 for countries with available data (Finland and Norway).
