# Supplementary material for: Greater mortality variability in the United States in comparison with peer countries
Source: Demogr Res. Author manuscript; Available in PMC 2020 Sep 16. (PMC7494211; doi:10.4054/demres.2020.42.36)
Supplement: DR_Supplementary_Material [file NIHMS1624231-supplement-DR_Supplementary_Material.pdf]

Appendix Figure A: High Sex-Specific U.S. interquartile, interdecile, and intercentile ranges of survivorship relative to peer countries, 1980-2018

Panel A: Interquartile range of survivorship in years

Females

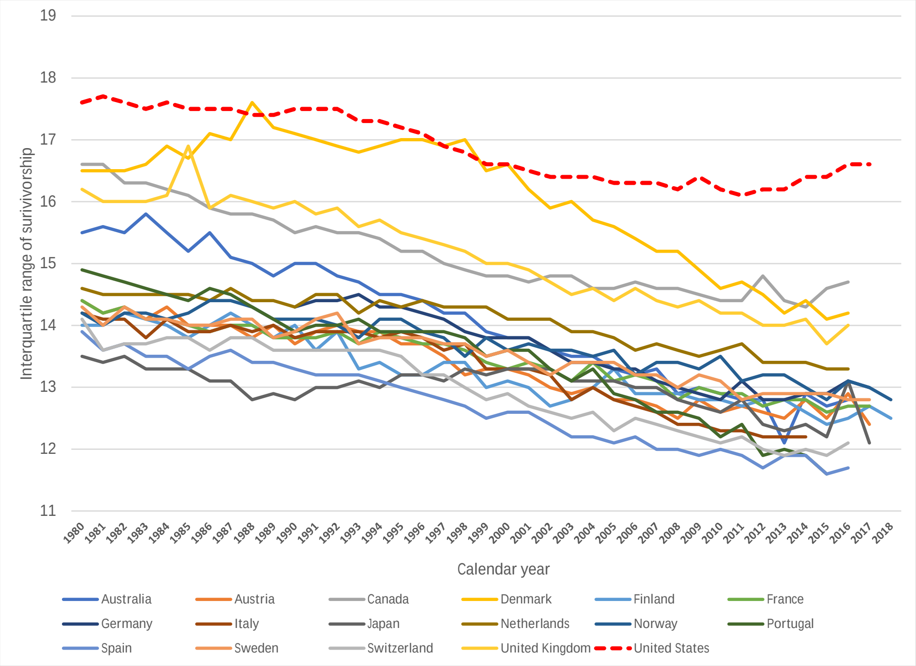

Males

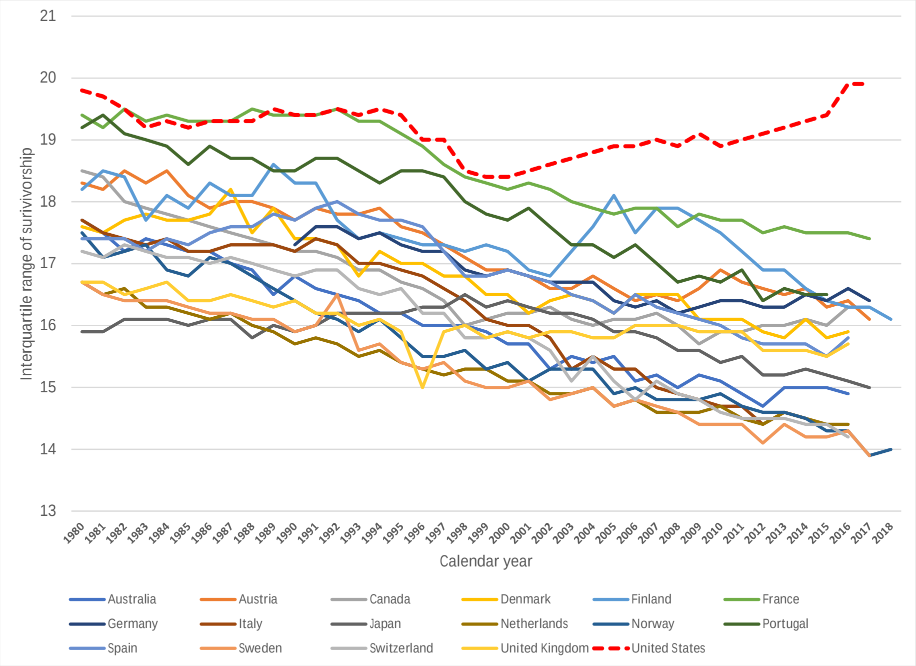

Panel B: Interdecile range of survivorship in years

Females

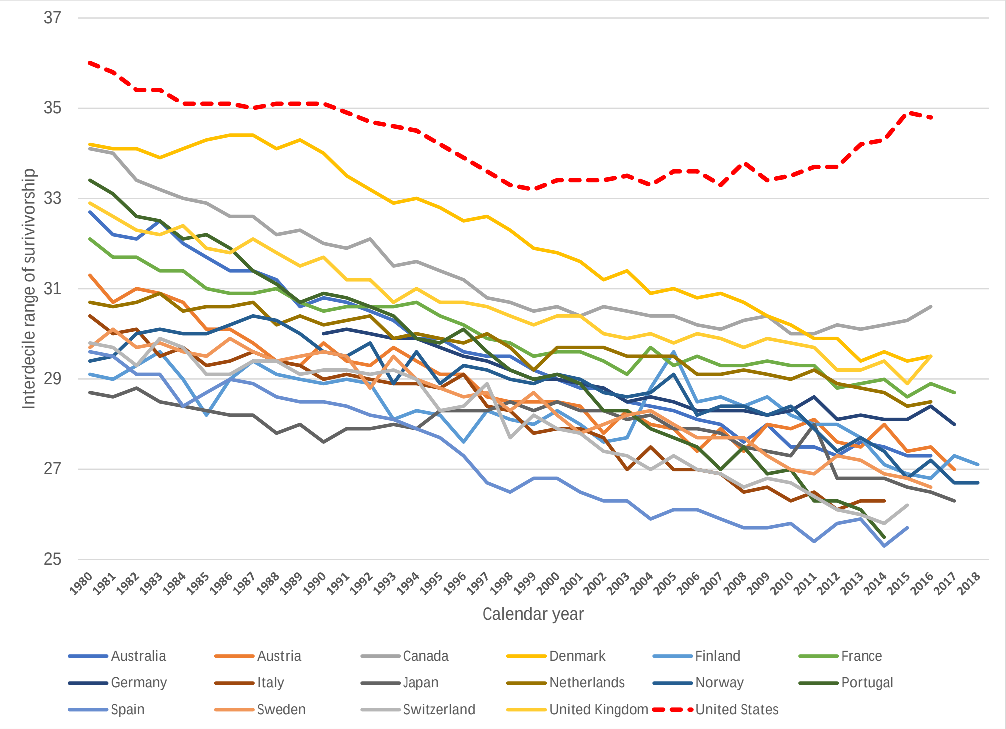

Males

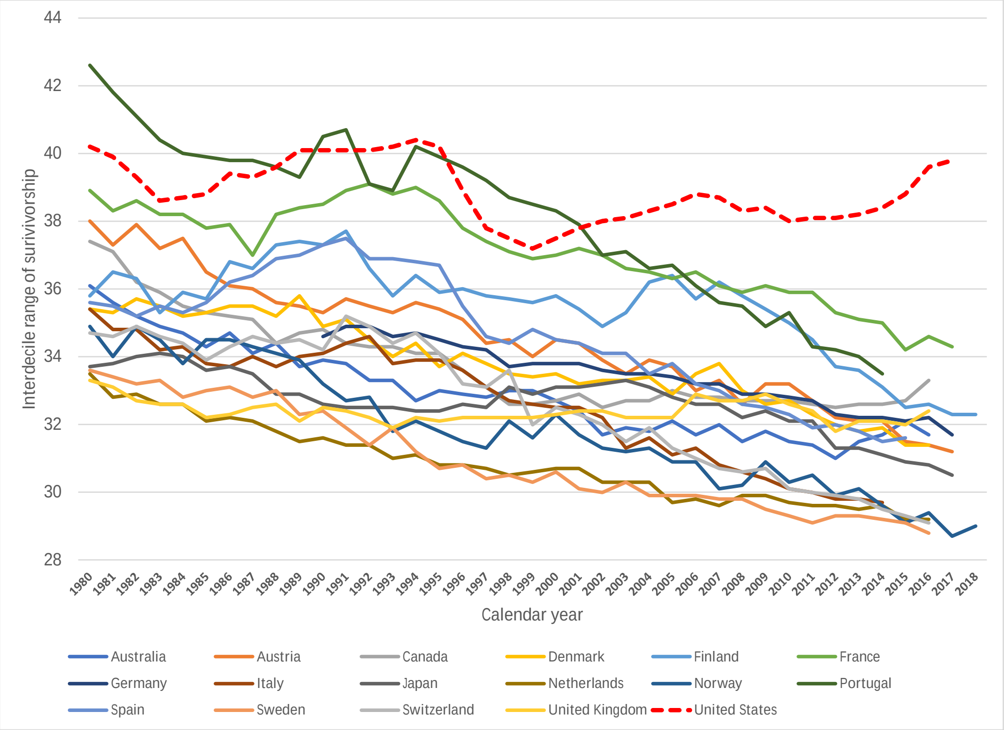

Panel C: Intercentile range of survivorship in years

Females

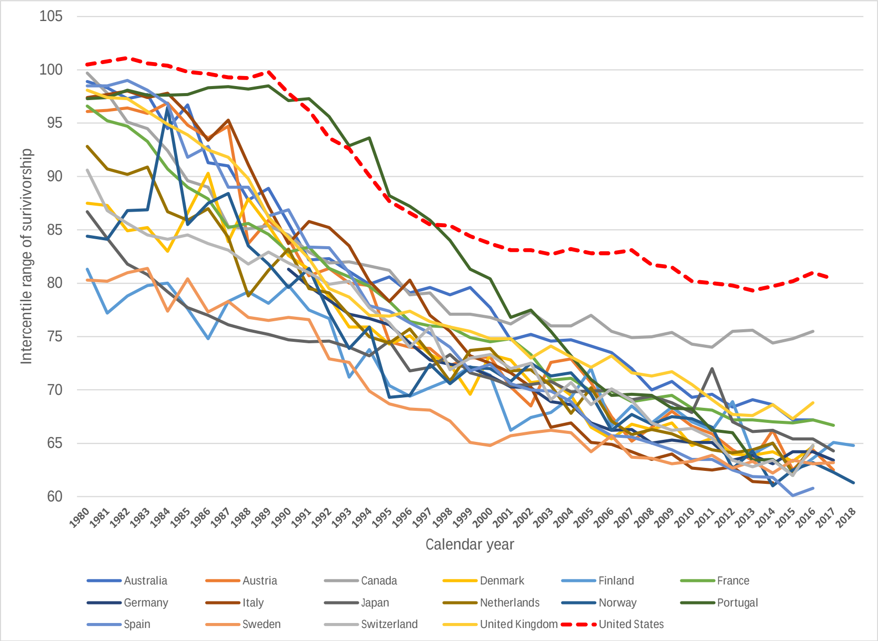

Males

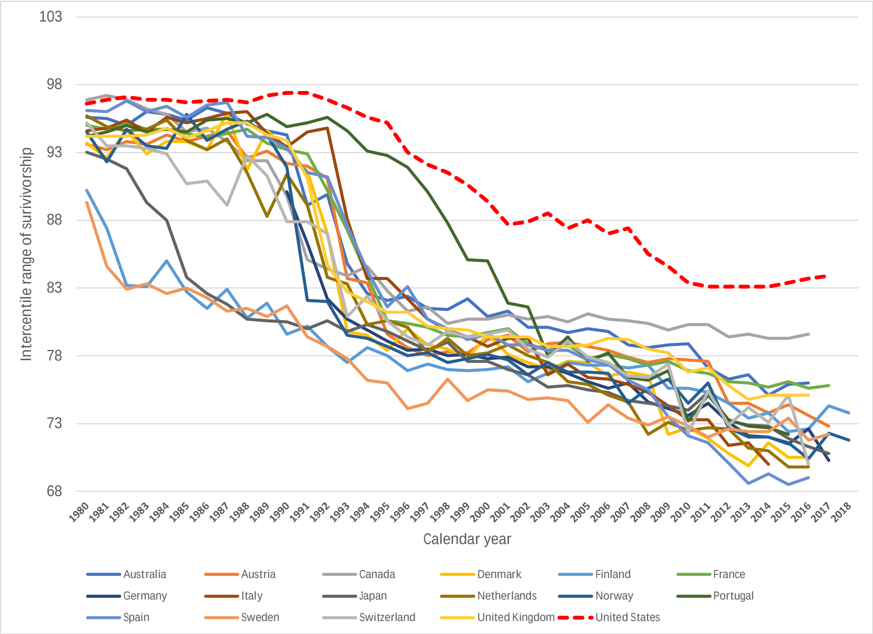

Notes: Total countries included in 1980 is 16, with Germany excluded. Total countries included in 2016 is 15, with Italy and Portugal excluded. The figure includes trends through 2018 for countries with available data (Finland and Norway).
Source: Derived from the Human Mortality Database (2020).
